# 

**Published:** 1893-01

**Authors:** 


### LIST OF CONTRIBUTORS TO VOLUME XIV.

ABBOTT, FRANK, New York.
ANDREWS, R. R., Cambridge, Mass.
BERGER, JOHN, Görlitz, Germany.
BISHOP, J. ADAMS, New York.
BLACK, G. V., Jacksonville, Ill.
BLAKEMAN, R. I., New York.
BÖDECKER, C. F. W., New York.
BONWILL, W. G. A., Philadelphia.
BRIGGS, CHARLES P., Boston, Mass.
BRIGGS, EDWARD C., Boston, Mass.
BROWN, GEO. V., Duluth, Minn.
BROWN, PETER, Montreal, Canada.
CARACATSANIS, D., Athens, Greece.
CASE, C. S., Chicago, Ill.
CAUSH, D. E., Brighton, Eng.
CURTIS, G. LENOX, New York.
DAVENPORT, W. S., Paris, France.
DENIS, DR., Paris, France.
EASTLAKE, ALBERT, New York.
EDDY, FORREST G., Providence, R. I.
FAUGHT, L. ASHLEY, Philadelphia.
FILLEBROWN, THOMAS, Boston, Mass.
FREEMAN, S., New York.
GIRDWOOD, JOHN, Edinburgh, Scotland.
GUILFORD, S. H., Philadelphia.
HOPKINS, F. S., Boston, Mass.
HOWE, J. MORGAN, New York.
HUGENSCHMIDT, A. C., Paris, France.
IVES, C. F., New York.
JACK, LOUIS, Philadelphia.
KELLEY, H. A., Portland, Me.
KIRK, EDWARD C., Philadelphia.
LADD, BABSON S., Boston, Mass.
LATHAM, VIDA A., Chicago, Ill.
LECAUDEY, E., Paris, France.
LORD, BENJAMIN, New York.
MCMANUS, JAMES, Hartford, Conn.
MILLS, G. A., New York.
OSGOOD, HAMILTON, Boston, Mass.
PAGE, EDWARD, Charlestown, Mass.
PALMER, S. B., Syracuse, N. Y.
PEIRCE, C. N., Philadelphia.

PERRY, SAFFORD G., New York.
RICHMOND, C. M., New York.
REILLY, JAMES A., Boston, Mass.
SCHREIER, EMIL, Vienna, Austria.
SHEPHERD, JAMES, Boston, Mass.
SIMONTON, W. S., Cameron, W. Va.
SMITH, P. T., Denver, Col.
SWIFT, AUTHUR L., New York.
STEWART, CARRIE M., Ann Arbor, Mich.
TALBOT, EUGENE S., Chicago, Ill.
THOMPSON, A. H., Topeka, Kansas.
THAYER, W. IRVING, Williamsburgh, Mass.
TRUMAN, JAMES, Philadelphia.
VAN ORDEN, L., San Francisco, Cal.
WAITT, J. E., Boston, Mass.
WHITNEY, J. M., Honolulu, H. I.
ZSIGMONDY, Otto, Vienna, Austria.

